# The positive association of branched-chain amino acids and metabolic dyslipidemia in Chinese Han population

**DOI:** 10.1186/s12944-016-0291-7

**Published:** 2016-07-25

**Authors:** Panpan Yang, Wen Hu, Zhenzhen Fu, Luning Sun, Ying Zhou, Yingyun Gong, Tao Yang, Hongwen Zhou

**Affiliations:** 1 Department of Endocrinology, the First Affiliated Hospital of Nanjing Medical University, Nanjing, 210029 Jiangsu Province China; 2 Department of Endocrinology and Metabolism, Huaian Hospital Affiliated to Xuzhou Medical College and Huaian Second People’s Hospital, Huaian, 223002 China; 3 Research Division of Clinical Pharmacology, the First Affiliated Hospital of Nanjing Medical University, Nanjing, 210029 Jiangsu Province China

**Keywords:** Branched-chain amino acids, Metabolic dyslipidemia, Triglyceride, HDL-C

## Abstract

**Background:**

It has been suggested that serum branched-chain amino acids (BCAAs) are associated with the incident, progression and prognostic of type 2 diabetes. However, the role of BCAAs in metabolic dyslipidemia (raised triglycerides (TG) and reduced high-density lipoprotein cholesterol (HDL-C)) remains poorly understood. This study aims to investigate 1) the association of serum BCAAs with total cholesterol (TC), TG, HDL-C and low-density lipoprotein cholesterol (LDL-C) and 2) the association between serum BCAAs levels and risk of metabolic dyslipidemia in a community population with different glucose homeostasis.

**Methods:**

Demographics data and blood samples were collected from 2251 Chinese subjects from the Huaian Diabetes Protective Program (HADPP) study. After exclusion for cardiovascular disease (CVD), serious hepatic or nephritic diseases and others, 1320 subjects remained for analysis (789 subjects with hemoglobin A1c (HbA1c) > 5.7, 521 with HbA1c ≤ 5.7). Serum BCAAs level was measured by liquid chromatography-tandem mass spectrometry (LC MS/MS). The association of BCAAs with lipids or with the risk of metabolic dyslipidemia was analyzed.

**Results:**

Elevated serum BCAAs (both total and individual BCAA) were positively associated with TG and inversely associated with HDL-C in the whole population. These correlations were still significant even after adjustment for confounding factors (*r* = 0.165, *p* < 0.001 for TG; and *r* = -0.126, *p* < 0.001 for HDL-C). For reduced HDL-C, we found higher odds risk (OR) of Valine (Val) in high HbA1c group than in the low one (OR = 1.055, *p* < 0.001 vs OR = 1.032, *p* = 0.059). Compared with that in the first quartile, the multivariable-adjusted OR (95 % CI) of the 4^th^ quartile of serum total BCAAs level for reduced HDL-C was 3.689 (2.325, 5.854) in high HbA1c group and 2.329 (1.284, 4.227) in low group, for raised TG was 3.305 (2.120, 5.152) and 2.972 (1.706, 5.176), and for metabolic dyslipidemia was 3.703 (2.261, 6.065) and 3.702 (1.877, 7.304), respectively (all *p* < 0.01).

**Conclusion:**

Elevated serum BCAAs level are positively associated with incident metabolic dyslipidemia. In addition, glucose homeostasis could play a certain role in BCAAs-related dyslipidemia.

## Background

Chronic metabolic diseases involving obesity and type 2 diabetes mellitus (T2DM) are pandemic worldwide. According to Guang Ning's report [1] in 2013, the prevalence of diabetes in adults was 11.6 % and pre-diabetes was 50.1 % among a representative sample of Chinese meaning up to 113.9 million Chinese adults with diabetes and 493.4 million with pre-diabetes. Diabetes is a cluster of metabolic syndrome featured by the deficiency of insulin secretion, insulin action or both, which causing metabolic disorders of nutrient substance including carbohydrate, protein, fat, water and electrolyte.

Obviously, it has been well documented that in patients with impaired glucose homeostasis are also commonly accompanied with metabolic dyslipidemia characterized by raised triglycerides (TG) and reduced high-density lipoprotein cholesterol (HDL-C) [2, 3]. They are main risk factors of cardiovascular disease (CVD) which leads to elevated mortality and heavy societal burden. Recently, many studies [4–7] have showed that elevated serum amino acids especially BCAAs level were associated with the onset of T2DM, which have provided a new potential interpretation for pathogenesis of T2DM, thus attracting worldwide attention.

Though elevations of BCAAs in obesity were first reported in the 1960s, until to 2009 Newgard [4] have successfully determined human serum BCAAs concentration by chromatography MS/MS (LC MS/MS) and have firstly investigated the association of BCAAs concentration with metabolic factors using metabonomics analysis. Remarkably, they found that BCAAs, aromatic amino acids (AAAs), C3 and C5 acylcarnitines, methionine, Glx (glutamate/glutamine) but not lipids-related metabolites had a stronger link to insulin resistance, which was also been demonstrated by other researches [5–9]. In another Framingham Offspring Study [10] Wang found that blood BCAAs and AAAs concentration were elevated before any alterations in insulin sensitivity could be measured by standard biochemical methods. What’s more, BCAAs and its metabolites have also been demonstrated as potent predictive factors for diabetes incidence, development and prognosis by amounts of literature [5, 11].

Since BCAAs played an important role in T2DM, how BCAAs participate in energy metabolism? BCAAs including leucine (Leu), isoleucine (Ile) and valine (Val) which are essential amino acids not only provide substrate and regulate proteins synthesis [12], but also act as signal molecules to control energy homeostasis involving glucose disposition and lipids metabolism [4, 13]. BCAAs catabolism can be divided into two steps. The first step is that mitochondrial isoform of branched-chain aminotransferase (mBCTA) reversibly catalyze BCAA into branched chain α-ketoacids (BCKA) by transamination. Next, BCKA are irreversible catalyzed by multienzyme mitochondrial branched-chain α‑ketoacid dehydrogenase complex (BCKDC) into corresponding coenzyme A which can be further oxidized for power in tricarboxylic acid (TCA) cycle. As key enzyme of the whole metabolism, the activity of BCKDC can be down-regulated by branched-chain α-ketoacid dehydrogenase kinase (BCKDK) and up-regulated by mitochondrial isoform of protein phosphatase 1 K (PPM1K).

As energy substance, Newgard [14] found that enhanced flux and the generation of BCAAs catabolic intermediates (propionyl CoA and succinyl CoA) could reduce the efficiency of oxidation of fatty acids and glucose, leading to accumulation of fatty acids. Metabolite of BCAAs could also divert into glycogen in liver and into fatty acids in adipose tissue. Interestingly, in a recent Japanese study [5] future risk of lifestyle-related diseases including metabolic syndrome and dyslipidemia more than diabetes could be all predicted by serum BCAAs levels.

However, the relevance of serum BCAAs to metabolic dyslipidemia remains poorly understood. Exploring this association could provide insight into potential mechanisms in the pathogenesis of metabolic dyslipidemia. Therefore, we aimed to 1) examine the association between serum BCAAs level and lipids (total cholesterol (TC), HDL-C, TG, low-density lipoprotein cholesterol (LDL-C)) and 2) investigate the risk of serum total BCAAs or individual BCAA for metabolic dyslipidemia, raised TG and reduced HDL-C in population with different HbA1c level.

## Results

### The demographics and characteristics of the study population

A total of 1310 participates (diabetes group (DB): 451, pre-diabetes group (impaired glucose regulation, IGR): 422 and normal glucose tolerance (NGT): 437) were recruited in this study, which in gender composition, women accounted for more than 60% (*p* < 0.001). The mean ages of DM, IGR and NGT groups were 60.27 ± 5.98, 59.33 ± 5.77 and 57.51 ± 5.95 years respectively (*p* < 0.001). Study population, anthropological measures, biochemical and chemical index were presented in Table 1. As shown in Table 1, DB group had a higher body mass index (BMI) (*p* < 0.001), waist circumstance (*p* < 0.001), fasting blood glucose (FBG) (*p* < 0.001) and HbA1c (*p* < 0.001) than IGR and NGT groups, which all had significant differences. The distribution of smoking was similar in three groups. While systolic blood pressure (SBP) had no difference in three groups, diastolic blood pressure (DBP) of NGT subjects was increased. Moreover, the ratio of metabolic dyslipidemia to subgroup number was higher in DB group than another two groups (*p* = 0.001).

**Table 1:** Demographic and laboratory characteristics of study subjects

	DM	IGR	NGT	*P* value	HbA1c > 5.7	HbA1c ≤ 5.7	*P* value
*N* = 1310	451	422	437		789	521	
Gender, male (%)	188 (41.7)^a^	150 (35.5)	130 (29.7)	0.001	304 (38.5)	164 (31.5)	0.01
Age	60.27 ± 5.98^a^#	59.33 ± 5.77^a^	57.51 ± 5.95	< 0.001	59.86 ± 5.77	57.82 ± 6.15	0.018
Smoking (%)	82 (18.2)	74 (17.5)	67 (15.3)	0.499	142 (18.0)	81 (15.5)	0.261
Height (m)	1.63 ± 0.71^a^	1.62 ± 0.07	1.62 ± 0.07	0.049	1.62 ± 0.07	1.62 ± 0.07	0.720
Weight (kg)	66.9 ± 11.40^a^#	64.98 ± 9.56^a^	62.78 ± 8.82	< 0.001	65.98 ± 10.74	63.28 ± 8.92	< 0.001
BMI (kg/m^2^)	25.07 ± 3.73^a^#	24.63 ± 2.96^a^	23.86 ± 2.9	< 0.001	24.91 ± 3.46	23.95 ± 2.85	< 0.001
Waist circumstance (cm)	85.91 ± 10.19^a^#	83.82 ± 9.30^a^	81.37 ± 8.24	< 0.001	85.01 ± 9.93	81.78 ± 8.36	< 0.001
SBP (mmHg)	140.17 ± 20.08	139.14 ± 18.86	140.35 ± 19.82	0.62	139.77 ± 19.42	140.10 ± 19.88	0.769
DBP (mmHg)	82.55 ± 14.56^a^	84 ± 12.56	84.99 ± 14.0	0.03	83.33 ± 13.74	84.59 ± 13.83	0.105
FBG (mmol/L)	7.37 ± 2.64^a^#	5.56 ± 0.57^a^	5.07 ± 0.32	< 0.001	6.59 ± 2.21	5.15 ± 0.52	< 0.001
HbA1c (%)	6.87 ± 1.48^a^#	6.05 ± 0.15^a^	5.32 ± 0.14	< 0.001	6.60 ± 1.12	5.31 ± 0.17	< 0.001
Amino acids							
Leu (μmol/L)	209.91 ± 43.72^a^	199.63 ± 41.61^a^	194.45 ± 39.95	< 0.001	205.25 ± 43.50	195.67 ± 39.72	< 0.001
Ile (μmol/L)	104.56 ± 21.53^a^	99.45 ± 21.85^a^	96.62 ± 23.10	< 0.001	102.22 ± 22.14	97.31 ± 22.48	< 0.001
Val (μmol/L)	282.72 ± 56.57^a^	267.00 ± 47.79^a^	260.33 ± 51.70	< 0.001	275.51 ± 53.19	262.13 ± 51.87	< 0.001
Total BCAAs (μmol/L)	597.19 ± 110.23^a^#	566.08 ± 99.08^a^	551.41 ± 103.50	< 0.001	582.98 ± 107.00	555.10 ± 102.76	< 0.001
Lipids							
TG (mmol/L)	2.28 ± 1.64^a^#	2.04 ± 0.96^a^	1.79 ± 1.06	< 0.001	2.20 ± 1.40	1.79 ± 1.01	< 0.001
TC (mmol/L)	5.17 ± 0.93	5.25 ± 0.85^a^	5.09 ± 0.81	0.026	5.23 ± 0.90	5.07 ± 0.80	< 0.001
LDL-C (mmol/L)	2.73 ± 0.74#	2.82 ± 0.69^a^	2.67 ± 0.67	0.005	2.78 ± 0.73	2.67 ± 0.66	0.002
HDL-C (mmol/L)	1.29 ± 0.40^a^	1.33 ± 0.36^a^	1.47 ± 0.63	< 0.001	1.31 ± 0.37	1.44 ± 0.61	< 0.001
Metabolic dyslipidemia							
N (%)	147 (32.6)^a^	119 (28.2)^a^	95 (21.7)	0.001	248 (31.4)	113 (21.7)	< 0.001

*p*-value: comparison among the three groups (left) or between the two groups (right); ^a^comparison with NGT; #comparison with IGR. IGR: subjects with impaired glucose regulation (IFG: impaired fasting glucose + IGT: impaired glucose tolerance), NGT: subjects with normal glucose tolerance. BMI: body mass index. SBP: systolic blood pressure. DBP: diastolic blood pressure. FBG: fasting blood glucose. HbA1c: hemoglobin A1c. Leu: leucine. Ile: isoleucine. Val: valine. BCAAs: branched-chain amino acids. TG: triglyceride. TC: total cholesterol. HDL-C: high-density lipoprotein cholesterol. LDL-C: low-density lipoprotein cholesterol

Interestingly, the serum individual BCAA and total BCAAs levels were significantly elevated in DB group (Leu: 209.91 ± 43.72, *p* < 0.001, Ile: 104.56 ± 21.53, *p* < 0.001, Val 282.72 ± 56.57, *p* < 0.001, BCAAs: 597.19 ± 110.23, *p* < 0.001).

### Elevated serum BCAAs was positively associated with TG and inversely associated with HDL-C

Serum BCAAs and lipids levels were examined in 1310 subjects. As seen in Table 2, serum Leu as well as Ile, Val and total BCAAs level all had a positive and significant correlation with serum TG level, even after adjustment of age, gender, BMI, SBP, DBP, FBG and smoking (Model 2 in Table 2). In contrast, total BCAAs and individual (Leu, Ile, Val) levels were negatively associated with HDL-C level even after adjustment for confounding factors listed above. Nevertheless, the link between serum total BCAAs and LDL-C or TC was not indicated in both two models. These results showed that BCAAs related to TG and HDL-C independently with fasting blood glucose.

**Table 2:** The cruel and adjusted Pearson correlations between serum BCAAs and lipids levels

	Model 1				Model 2			
	Leu	Ile	Val	BCAAs	Leu	Ile	Val	BCAAs
TG	*r* = 0.187^**^ *p* = 0.000	*r* = 0.202^**^ *p* = 0.000	*r* = 0.196^**^ *p* = 0.000	*r* = 0.216^**^ *p* = 0.000	*r* = 0.144^**^ *p* = 0.000	*r* = 0.160^**^ *p* = 0.000	*r* = 0.143^**^ *p* = 0.000	*r* = 0.165^**^ *p* = 0.000
HDL-C	*r* = -0.182^**^ *p* = 0.000	*r* = -0.187^**^ *p* = 0.000	*r* = -0.104^**^ *p* = 0.000	*r* = -0.164^**^ *p* = 0.000	*r* = -0.151^**^ *p* = 0.000	*r* = -0.156^**^ *p* = 0.000	*r* = -0.064^**^ *p* = 0.021	*r* = -0.126^**^ *p* = 0.000
LDL-C	*r* = 0.044 *p* = 0.114	*r* = 0.025 *p* = 0.358	*r* = -0.023 *p* = 0.397	*r* = 0.010 *p* = 0.716	*r* = 0.052 *p* = 0.063	*r* = 0.040 *p* = 0.148	*r* = -0.023 *p* = 0.399	*r* = 0.016 *p* = 0.553
TC	*r* = -0.001 *p* = 0.964	*r* = -0.046 *p* = 0.096	*r* = 0.014 *p* = 0.617	*r* = -0.003 *p* = 0.909	*r* = 0.023 *p* = 0.405	*r* = -0.012 *p* = 0.669	*r* = 0.028 *p* = 0.321	*r* = 0.021 *p* = 0.453

*N* = 1310. All amino acid levels were log-transformed. Model 1: the cruel analysis. Model 2: adjusted for age, gender, SBP, DBP, FBG, BMI and smoking.

** p<0.01

### The risk of serum BCAAs levels for metabolic dyslipidemia and its components in low and high HbA1c groups

**Fig. 1:**
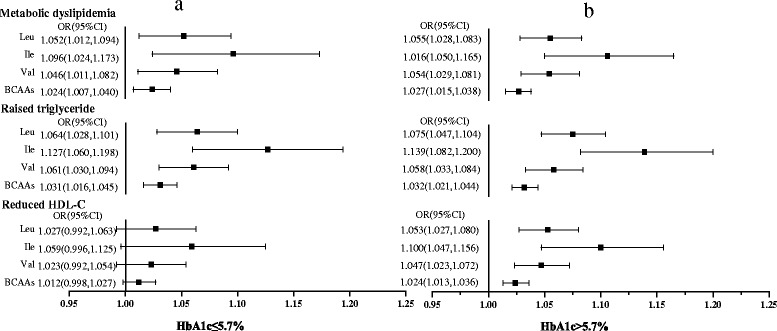
The cruel logistic regression model of BCAAs with metabolic dyslipidemia and its components

**Fig. 2:**
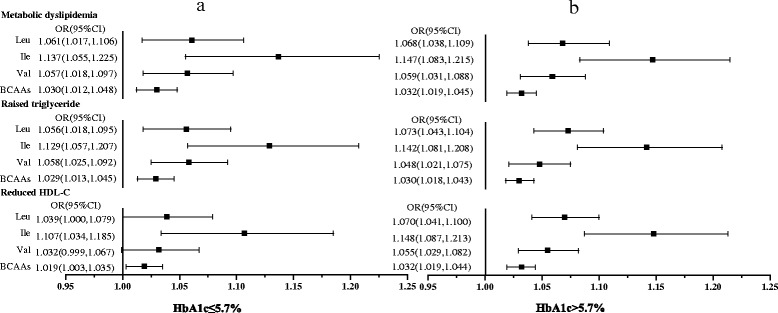
The adjusted logistic regression model of individual BCAA with metabolic dyslipidemia and its components. Adjusted for age, gender, SBP, DBP, FBG, BMI and smoking

To investigate the association of BCAAs with metabolic dyslipidemia in different glucose metabolism population, the whole population was divided into two parts: HbA1c > 5.7 % and HbA1c ≤ 5.7 groups. As depicted in Table 1, serum Leu, Ile, Val and total BCAAs concentrations were higher in HbA1c > 5.7 % group than those in HbA1c ≤ 5.7 group. Logistic regression model was used to analyze the risks of serum individual or total BCAAs levels for metabolic dyslipidemia and its components in low and high HbA1c groups. As shown in (Fig. 1 a and b), in the cruel model, there were no significant differences between the risks of individual BCAA for metabolic dyslipidemia in the two groups. The risks of individual BCAA for reduced HDL-C were significant in the high HbA1c group, while risks in the low group weren’t significant. To eliminate the effects of confounding factors, age, gender, BMI, SBP, DBP, FBG and smoking were adjusted. In the adjusted regression model (Fig. 2a, b), we found 1.055 increased risk of Val level for reduced HDL-C in high HbA1c group compared to the low group. There were no differences in the risks of Leu or Ile for raised TG, reduced HDL-C and metabolic dyslipidemia between the high and low HbA1c groups.

**Fig. 3:**
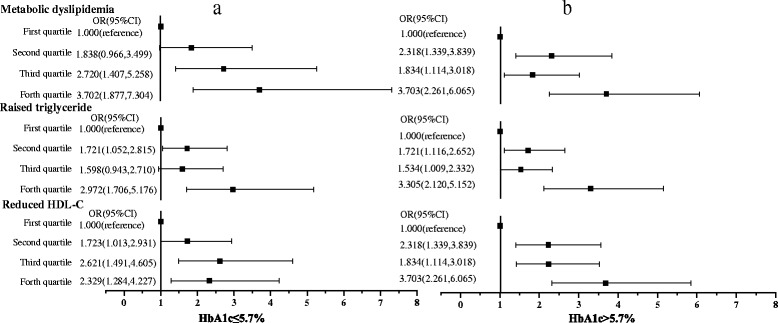
The odds ratios for incidence of metabolic dyslipidemia and its components. The 25^th^, 50^th^, 75^th^ percentiles were used as cut-off points for serum total BCAAs level. Adjusted for age, gender, SBP, DBP, FBG, BMI and smoking

For serum total BCAA level, each standard deviation (SD) increment was separately associated with a 3.2 % and 1.9 % increased odds of reduced HDL-C in high and low HbA1c groups (Fig. 2a, b). Compared with that in the first quartile, the multivariable-adjusted OR (95 % CI) of the 4th quartile of serum total BCAAs level for reduced HDL-C was 3.689 (2.325, 5.854) in high HbA1c group and 2.329 (1.284, 4.227) in low group, for raised TG was 3.305 (2.120, 5.152) and 2.972 (1.706, 5.176), and for metabolic dyslipidemia was 3.703 (2.261, 6.065) and 3.702 (1.877, 7.304), respectively (all *p* < 0.01) (Fig. 3a, b).

## Discussion

Our data showed that in Chinese population, compared to NGT subjects, individuals with T2DM or high HbA1c level (HbA1c > 5.7 %) had higher serum BCAAs level, and in the whole population of this study elevated serum BCAAs was positively associated with TG and inversely associated with HDL-C independent of FBG. Moreover, there was no difference in the risks of serum individual BCAA or total BCAAs for metabolic dyslipidemia between the high and low HbA1c groups. These indicated that 1) serum BCAAs level was correlated to diabetes status or HbA1c level, 2) increased BCAAs might have impact on the process of lipids metabolism, and 3) this impact could be independent of glucose metabolism status.

We estimated the serum BCAAs levels in a relative large population by LC MS/MS method and the data showed that patients with diabetes or pre-diabetes had a significantly elevated serum BCAAs levels which was consistent with other studies [8, 15, 16]. The mechanism of this phenomenon has not been elucidated. Increased protein intake, uncontrolled of protein turnover and disturbance of BCAAs per se catabolism had been thought to be the main contributors. High protein or BCAA ingestion increased serum BCAAs concentration in a short-term dietary intervention [17, 18], which seems to be able to partly explain insulin resistance induced by high protein intake, while another study [19] revealed that high protein or BCAAs uptake could be associated with decreased risk of diabetes in 13525 cohort in Japanese resident. Insulin could be responsible for protein synthesis [20]. Protein breakdown or turnover could be enhanced by impaired insulin signaling, leading to accumulation of BCAAs [21]. Moreover, decreased activities or contents of BCAAs oxidative enzymes have well been documented both in obese or diabetic rodents and humans [22, 23]. Bajotto G [24] found a decreased activity and contents of hepatic BCKDC, a rate-limiting enzyme of BCAAs catabolism in a T2DM rat model. Others also observed [23, 25] that Methyl-malonate-semialdehyde dehydrogenase and propionyl-CoA carboxylase β which are required for Val and Ile metabolism before entry into TCA cycle were lower in obese and insulin resistance subjects than those in lean ones. Recently, the vital role of adipose tissue on circulating BCAAs level had been firstly elucidated by Herman MA [26], she found that transplantation of normal adipose tissue into mice defective in peripheral BCAA metabolism reduced circulating BCAA levels by 30 % (fasting)-50 % (fed state), which indicated high circulating BCAAs level could be partly leaded by decreased oxidation of BCAA in adipose tissue in obese or diabetes subjects. Another study [27] also found that elevated serum BCAAs level could be caused by liver and adipose tissue.

We demonstrated that there were significant associations between BCAAs and TG or HDL-C, but there was no relationship between BCAAs and TC or LDL-C, which proclaimed that BCAAs could present the lipids-related CVD risks to some extent by affecting lipids metabolism. The FIELD randomised trial [28] evaluated the risk of CVD on diabetes with or without metabolic syndrome (MS) and found that patients absent of MS had a lower risk of future CVD events, hypertension and dyslipidemia. A recent study [8] also elucidated a link between serum BCAAs level and vascular deterioration in a population with high HbA1c. However, no precise mechanism regarding these links had been investigated. In our study, there were significant differences in the risks of serum individual or total BCAAs for metabolic dyslipidemia both in high (HbA1c > 5.7 %) and low HbA1c groups, that means serum BCAAs were risk factors of metabolic dyslipidemia in the whole population, which was consistent with reports that seven amino acids including BCAAs were associated with an increased risk of hypertriglyceridemia not only in diabetes subjects but also in non-diabetes ones demonstrated by K. Suhre after a 7-years follow-up [9]. Similarly, the Japanese study [5] also exhibited a predictive ability of BCAAs to dyslipidemia after four years. This link still existed after adjustment for age, gender, BMI, waist circumference, SBP, DBP, smoking and FBG, indicating BCAAs could affect lipids metabolism before elevated blood glucose or insulin resistance could be measured by clinical methods. Our results not only add a new evidence to confirm researches above again, but also put forward a question that whether and how impaired glucose metabolism put impact on the risks of BCAAs for metabolic dyslipidemia. Results exhibited in Fig. 2b illuminated that in HbA1c > 5.7 % group, the risks of serum BCAAs for metabolic dyslipidemia, raised TG and reduced HDL-C were all significant, which indicated that glucose and BCAAs could synergize the pathogenesis of metabolic dyslipidemia and its components.

Adipose tissue and liver could be the main sites for interaction of BCAAs and lipids metabolism. As mentioned above, adipose tissue played an important role in BCAAs homeostasis [26, 29]. Adversely, BCAAs metabolism was also of great significance to adipose cells differentiation and lipogenesis [30]. The concentrations of BCAAs and its intermediates comprising C3 and C5 acylcarnitine were higher in obese [4], diabetic or high HbA1c individuals [8]. Our data also showed elevated BCAAs in patients with high HbA1c, unfortunately, we didn't examine serum C3 and C5 acylcarnitine levels which reflected the pool of propionyl-CoA, substrate for fatty acids biosynthesis. Data from Halama A's study [31] showed that 3-hydroxy-3-methylglutaryl-CoA, a byproduct of Leu degradation might serve as a substrate for cholesterol biosynthesis, and that degradation of Ile might be correlated with odd fatty acids. What's more, Leucine deprivation [32] in male C57BL/6 J mice could increase lipolysis and enhance expression of β-oxidation genes, while the expression of lipogenic genes and the activity of fatty acid synthase in white adipose tissue were reduced. Therefore, we supposed that enhanced fatty acids or cholesterol biosynthesis ability of adipose tissue could be a reason for metabolic dyslipidemia. In low HbA1c group, we found the risk of serum BCAAs for metabolic dyslipidemia was also significant. This might be explained by that a part of diabetic patients with rigorous glucose control were assigned to the low group.

Liver seems to be another candidate organ responsible for the association between BCAAs and metabolic dyslipidemia due to its special role in lipids metabolism. It has been demonstrated that BCAAs correlated with nonalcoholic fatty liver disease (NAFLD) which was common morbidity of insulin resistance, obesity and T2DM [4, 29, 33]. On the one hand, high BCAAs concentration could arouse insulin resistance in insulin-action organs, particularly in hepatic cells [34, 35]. Increased gluconeogenesis and hepatic glycogen export impaired the hepatic lipids homeostasis, therefore leading to accumulation and deposition of triglycerides and other fatty acids [36, 37]. When exceed the lipids disposal ability of liver, excessive lipids accumulated in circulation bringing about dyslipidemia. On the other hand, as molecular regulators of energy metabolism, BCAAs could directly participate in and damage hepatic TCA cycle which alleviated β-oxidation of fatty acids, and this maybe a potential reason for NAFLD and metabolic dyslipidemia [33].

However, BCAAs is widely used in clinic to improve malnutrition of liver disease [38–40] due to stimulation of protein synthesis [41]. Thus, we need to pay more attention on serum BCAAs level in individuals with hyperlipidemia, T2DM or CVD, and individuals accepting BCAAs treatment with liver cirrhosis or other chronic hepatopathy.

## Conclusion

In conclusion, our results illustrated that there was an elevated level in serum individual or total BCAAs in patients with diabetes or high HbA1c level and elevated serum BCAAs was positively associated with TG and inversely associated with HDL-C in a Chinese Han community population. These provide a new potential explanation for serum BCAAs predictive ability of CVD events. However, further investigation need to be done for predictive ability of BCAAs on dyslipidemia, CVD or other metabolic diseases.

## Methods

### Study population

In the present study, 2251 participates aged 40 to 79 years were recruited who were retiree and accepted annual routine health examinations between August 2014 and December 2014 at the health examination center of Huaian Second Hospital, Affiliated Hospital of Xuzhou Medical College in Huaian (Jiangsu, China). Exclusion criteria included 1) CVD, 2) serious hepatic or nephritic diseases, 3) peripheral artery atherosclerosis, and 4) tumors. In addition, 6 subjects were excluded because of missing HbA1c value, leaving a total of 1320 subjects (455 with diabetes (DB), 426 with pre-diabetes (IGR) and 439 with NGT). Written informed consent was obtained from all participants who were informed in detail about the objectives and procedures of the study before. This cross-sectional study was rooted in Huaian Diabetes Prevention Program (HADPP) (ChiCTR-TRC-14005029) which was approved by the Huaian Second Hospital Ethics Committee, Xuzhou Medical University, China.

### Date collection

Demographics data (age, gender, ethnicity) were collected at their first visiting. Also, height, weight, SBP and DBP were measured as described before [42]. BMI was calculated as weight (kg) divided by height squared (m^2^). Diabetes and IGR were defined based on 2012 ADA criteria [43]. Metabolic dyslipidemia was defined as a serum TG > 1.70 mmol/L, and HDL-C < 1.04 mmol/L in men or HDL-C <1.3 mmol/L in women [3].

### Determination of serum BCAAs level and other parameters

Venous blood samples were obtained early morning after overnight fasting and then centrifuged to gain serum for measurement of BCAAs, lipids and others. Selective hydrophilic interaction LC MS/MS we previously reported [44] was used to quantify serum BCAAs level. Briefly, 0.05 mL serum sample and 0.05 mL water were mixed with 0.9 mL acetonitrile (containing mixed internal standards working solution 3 μg/mL, each), and the mixture were centrifuged at 16000 rpm for 15 min (4 °C) and the supernatant (200 μL) was transferred to autosampler vial. HbA1c was measured by high performance liquid chromatography (Variant II and D-10 Systems, Bio-Rad Laboratories Inc., Hercules, CA, USA), and TC, TG, LDL-C, HDL-C was determined which had reported before [42].

### Statistical analyses

The categorical variables in this study are presented as numbers (%) while continuous variables exhibited normal distributions, which are summarized in terms of means ± SD. For analysis, the subjects were divided into four groups based on serum total BCAAs level using the 25^th^, 50^th^, 75^th^ percentiles as cut-off points. Comparisons between groups were made using the one-way analysis of variance (ANOVA) or *t*-test for continuous data and the chi-square test for categorical data. Correlations were assessed using partial correlation coefficients before and after adjusting for confounding factors. Logistic regression was used to identify the factors associated with metabolic dyslipidemia and its components. All presented P-values are two-tailed, and a P-value < 0.05 was considered to indicate statistical significance. Analysis were performed using SPSS software, version SPSS 16.0 (SPSS Inc., Chicago, IL, USA).

## Abbreviations

AAs, Aromatic amino acids; ADA, American Diabetes Association; one-way ANOVA, One-way analysis of variance BCAAs, Branched-chain amino acids; BCKA, Branched chain α-ketoacids; BCKDC, Multienzyme mitochondrial branched-chain α‑ketoacid dehydrogenase complex; BCKDK, Branched-chain α‑ketoacid dehydrogenase kinase; BMI, Body mass index; CVD, Cardiovascular disease; DBP, Diastolic blood pressure; DB, Diabetes group; FPG, Fasting plasma glucose; HADPP, Huaian Diabetes Protective Program study; HbA1c, Hemoglobin A1c; HDL-C, High-density lipoprotein cholesterol; IGR, Impaired glucose regulation; Ile, Isoleucine; LC MS/MS, Chromatography MS/MS; LDL-C, Low-density lipoprotein cholesterol; Leu, Leucine; mBCTA, Mitochondrial isoform of branched-chain aminotransferase; MS, Metabolic syndrome; NAFLD, Nonalcoholic fatty liver disease; NGT, Normal glucose tolerance; PBG, Postprandial blood glucose; PPM1K, Mitochondrial isoform of protein phosphatase 1 K; SBP, Systolic blood pressure; SD, Standard deviation; T2DM, Type 2 diabetes Mellitus; TC, Total cholesterol; TCA, Tricarboxylic acid cycle; TG, Triglyceride; Val, Valine.
